# Clinical and Microbiological Performances and Effects on Lipid and Cytokine Production of a Ceruminolytic Ear Cleaner in Canine Erythemato-Ceruminous Otitis Externa

**DOI:** 10.3390/vetsci9040185

**Published:** 2022-04-13

**Authors:** Fabien Moog, Johanna Mivielle, Jessie Brun, Mirabela Oana Dumitrache, Nicolas Amalric, Line-Alice Lecru, Charline Pressanti, Jevgenija Kondratjeva, Daniel Combarros, Oscar Fantini, Marie Christine Cadiergues

**Affiliations:** 1 Department of Clinical Sciences, Université de Toulouse, ENVT, 31076 Toulouse, France; fabien.moog@envt.fr (F.M.); johanna.mivielle@gmail.com (J.M.); jessie.brun@envt.fr (J.B.); l.lecru13@hotmail.com (L.-A.L.); charline.pressanti@envt.fr (C.P.); jevgenija.kondratjeva@envt.fr (J.K.); daniel.combarros@envt.fr (D.C.); 2 Department of Parasitology and Parasitic Diseases, Faculty of Veterinary Medicine, University of Agricultural Science and Veterinary Medicine Cluj-Napoca, 400372 Cluj-Napoca, Romania; mirabela.dumitrache@usamvcluj.ro; 3 QIMA Life Sciences, 31670 Labège, France; nicolas.amalric@qima.com; 4 INFINITy, Université de Toulouse, Inserm, CNRS, UPS, 31059 Toulouse, France; 5 Laboratoire Vétoquinol, 37 rue de la Victoire, 78009 Paris, France; oscar.fantini@vetoquinol.com

**Keywords:** dog, ear cleanser, erythemato-ceruminous otitis, ear lipids, cytokines, ear infection

## Abstract

Erythemato-ceruminous otitis externa (ECOE) is the most common type of otitis in dogs and is generally associated with bacterial and/or yeast infections. The performance of an ear cleaner was assessed over two weeks in canine ECOE, associated with a mild or moderate secondary infection, in a prospective open-label study. Forty ear canals with ECOE that did not receive any type of aural treatment and were not cleaned for 7 days were included. Pruritus (PS), 0–3 Otitis Index Score (OTIS-3) and 0–4 scale cytology (CYTO) scores were assessed on Day (D) 0, D7 and D14. Concentrations of a panel of 13 cytokines on the ear canal surface and the lipid profile of the exudate were measured on D0 and D14. From D0 to D12 or D13, the dogs’ ears were cleaned daily if the secretion score (SEC) was 3/3, every second day if the score was 2/3 and every third day if the score was 1/3. PS, OTIS-3, SEC and CYTO were significantly lower on D7 compared to baseline (−40%, −31%, −36%, −34%, respectively; *p* < 0.0001). The same parameters decreased further on D14 (−60%, −53%, −61%, −73%, respectively; *p* < 0.0001) and amounts of interleukin 8 and chemokine KC-like were also reduced compared to baseline (−45%, *p* < 0.01; −36%, *p* = 0.3, respectively). The lipid profile was also modified, with a decrease in free lipids and an increase in bound lipids.

## 1. Introduction

Canine otitis externa is a condition frequently seen in small animal veterinary practices with an approximate incidence of 10–15% [1]. Erythematous otitis externa (EOE) is the most common type, while suppurative otitis is more rare [2]. The causes of otitis externa are divided into predisposing factors that will increase the probability of developing the disease, primary factors that trigger the disease, secondary factors that aggravate the disease and perpetuating factors that prevent resolution [3]. Bacterial and yeast infections are important secondary factors that complicate and aggravate the disease [4]. Most acute cases are readily managed with topical products combining an antibiotic, an antifungal and a corticosteroid [5]. However, otitis externa frequently recurs, due to the persistence or recurrence of causative factors, whether local (e.g., polyp, tumour) or general (e.g., atopic dermatitis, seborrhoea) [1,6]. The ongoing cycle of infection and inflammation can eventually lead to stenosis, thickening/mineralisation of the ear canal, impaired drainage of secretions and proliferation of infectious agents such as yeasts and bacteria with selection of resistant organisms [1,6,7].

Proper cleansing of the ear is very valuable, as the exudate not only impairs otoscopic examination but also prevents effective therapy, as pus and inflammatory debris can inactivate some medications and prevent contact with the epithelial lining of the ear [5]. Cleansing removes microbes, bacterial toxins, cellular debris and free fatty acids, thereby reducing inflammation [8]. It is also the primary treatment when epithelial migration fails, as it prevents the build-up of cerumen and debris that can change the ear canal environment and promote secondary bacterial or yeast infection [1,6]. Various ear cleaners are routinely used, and some have been shown to have an antiseptic effect, either on yeasts or bacteria [9,10,11,12]. This is an interesting concept given the increase in multiple-antimicrobial-resistant bacteria and yeasts likely due to selective pressure caused by excessive/inappropriate antimicrobial use [13,14]. Unlike antibiotics, antiseptics act at the site of application and are less likely to promote resistance when used at high concentrations [15,16], although antibiotic resistance can emerge after exposure of various Gram-negative and Gram-positive species to sublethal concentrations of certain biocides such as benzalkonium chloride, chlorhexidine or triclosan [17,18,19]. This aspect has already been taken into account, as some ear cleaners have added antiseptic agents and can therefore find their place in the management of ECOE where topical steroids are contraindicated or have side effects [8,10,20]. However, a previous study showed that in vitro antibacterial effects of ear cleaners vary [20].

The main objective of this study was to evaluate in vivo the effect of an ear cleaner on the infectious and inflammatory components of canine ECOE, as well as its clinical performance. The secondary objectives were to advance our knowledge of *(i)* the bacterial flora involved in nonsuppurative otitis and *(ii)* the cytokines and the lipids expressed/found in ECOE.

## 2. Materials and Methods

### 2.1. Ethics

The animal experiments were approved by the Sciences et Santé Animales (SSA–Ecole Nationale Vétérinaire Toulouse) N°115 Ethics Committee (Approval No. SSA_2019_003). Written consent of the dogs’ owners was obtained prior to the study.

### 2.2. Animals

Ear canals of otherwise healthy client-owned dogs with ECOE were included in the study. The inclusion criteria were animals in good general health and the absence of systemic signs, as well as any other skin abnormalities as confirmed by a general physical examination by a licensed veterinarian. Enrolled dogs had bilateral or unilateral ECOE with a cytological score of 2 to 4 out 4 (2: <5 microbes on all high-power fields (HPF); 3: 5–50 microbes on all HPF; 4: >50 microbes on all HPF) [21,22] with no inflammatory cells (nonpurulent otitis) and an 0–3 Otitis Index Score (OTIS-3) score [23] of more than 0 for secretions but 0 for ulcers. The hyperplasia and erythema scores were not taken into account for inclusion. 

Exclusion criteria were animals whose ears had been cleaned in the past seven days and had received auricular treatment less than seven days prior to inclusion (products with prolonged persistence needed a withdrawal time of at least 21 days). Dogs who had received a systemic antimicrobial treatment less than seven days prior to inclusion (except cefovecin with a drug withdrawal time of at least 21 days) were excluded. Animals in poor body condition or individuals with certain parasitic infestations such as demodicosis or *Otodectes cynotis* infestation were also excluded. Enrolled dogs did not receive any auricular treatment or systemic antibiotic/antifungal treatment for the duration of the study. No ear cleaner was used other than the product being tested. Authorised treatments included nutritional supplements and medications that had not been modified over the last three months (including glucocorticoids, oclacitinib, ciclosporin), antiparasitic drugs, antiseptic and antiseborrheic shampoos and drugs that do not influence the cutaneous barrier. No change in food or in the environment occurred during the study.

### 2.3. Design of the Study

This was a monocenter, prospective open 14-day study. The experimental unit was the ear canal. The ear cleaner selected contained ethoxydiglycol, capric glycerides, isopropyl alcohol, calendula, tromethamine, glycerin and lipacids (Sonotix^®^, Vétoquinol SA, Lure, France). This product is intended for the routine cleaning of the ears of dogs and cats and for cleaning before the application of an auricular treatment and is labelled for usage in companion animals. 

The cleaning frequency was based on the secretion score recorded on Day (D0) [23]. An ear canal with a score of 3 was cleaned every day (from D0 to D13), every second day (D0, D2, D4, D6, D8, D10 and D12) with a score of 2 and every three days (D0, D3, D6, D9 and D12) with a score of 1. The cleaning was performed by the owner, after demonstration by the investigator. To prevent contamination of the ear cleaner, the owner was instructed to fill the external canal with the product without touching the skin with the tip of the bottle, then to gently massage the base of the canal for one minute and to let the dog shake its head before wiping the residue off with paper towels. The tip of the bottle was cleaned with a wipe soaked with 70 °C alcohol. Each bottle was weighed before the study and at the end of the study to determine the amount used (density 1.006 g/mL).

A total of three examinations were performed: before the study (D0), in the middle (D7) and at the end of the project (D14). All visits and examinations were performed before the application of the ear product. Each visit comprised a complete physical examination, exudate sampling, auricular pruritus scoring, OTIS-3 scoring using a video otoscope and a cytological examination with scoring.

### 2.4. Clinical Assessment

The ears were examined by the same junior scholar specifically trained in canine otology (J.M.) with a video otoscope (Dailyscope, Optomed, Les Ulis, France) and OTIS-3 was used to evaluate erythema, hyperplasia, secretions and ulcers, each parameter was scored out of 3 giving a total score out of 12 [23]. The ulcer parameter had to remain at 0 for the duration of the study. Tympanic membrane integrity was assessed during video-otoscopy, whenever possible.

Aural pruritus was assessed by the owner using a visual analogue scale out of 10. The owner was asked to fill out a satisfaction questionnaire about the product (smell, ease of use, overall satisfaction, each criterion on a 0–4 scale) at each follow-up visit.

### 2.5. Ear Canal Exudate Cytology and Microbial Cultures

Cytology was performed on D0, D7 and D14 by collecting exudates with a standard dry nonsterile cotton swab from the junction of the vertical and horizontal ear canals. The collected material was rolled onto a glass slide, air-dried and stained using the RAL^®^ 555 kit (RAL Diagnostics, Martillac, France) stain with dips lasting 5 s each time, according to the manufacturer’s recommendation. The slides were rinsed with tap water and blot dried on absorbent paper. The cytological score was applied by a board-certified veterinary dermatologist (M.C.C.) to each slide by evaluating 10 areas at HPF (×1000). Germs included bacteria (cocci and rods) and yeasts. A score of 0 was attributed to the total absence of germs, a score of 1 for a few germs on a few fields, 2 for less than five germs in each field, 3 for 5–50 germs in each field and 4 for >50 germs in all fields [21,22]. The amount of each type of microbe was assessed individually, and a global cytological score was given according to the above-mentioned guidelines.

On D0 and D14, a sterile swab sample from each affected ear was collected (Eswab^®^, Copan s.p.a., Brescia, Italy), and bacterial culture (aerobic and anaerobic) and sensitivity testing, as well as for fungal culture, were performed. The samples were transferred to the laboratory at room temperature on the same day. For bacterial culture, inoculation was carried out on three culture media (BBL™ CHROMagar™ Orientation/Gélose Columbia ANC, Chocolate Polyvitex + Bacitracine, Schaedler agar; Becton Dickinson GmbH, Heidelberg, Germany). Identification was carried out by mass spectrometry (Bruker France SAS, Wissembourg, France). Fungal cultures were grown on Sabouraud dextrose agar.

### 2.6. Lipid Contents of the Ear Canal Exudate

On D0 and D14, exudate was collected by gentle rubbing with two dry sterile swabs (MW112 Dryswab, Medical Wire & Equipment, Corsham, UK). The swab heads were then cut from the handle, placed in dry Eppendorf tubes and stored at −20 °C until lipid analysis. Neutral lipids (free and bound) were analysed by gas chromatography combined with mass spectrometry (GC/MS) after Bligh and Dyer liquid/liquid extraction [24]. A semi-quantitative analysis was performed. Results are expressed in area ratio vs. internal standard.

### 2.7. Cytokine Content of the Ear Canal Exudate

Lastly, on D0 and D14, two swabs (MW112 Dryswab, Medical Wire & Equipment, Corsham, UK) with prior soaking with an aqueous nonionic surfactant solution (QIMA Life Sciences proprietary method) to ensure optimal subsequent protein extraction, were successively introduced in the ear canal and rubbed over the whole surface of the canal for five seconds. The swab heads were then cut from the handle, placed in dry Eppendorf tubes and stored at −20 °C until cytokine analysis. Luminex^®^ technology was used with a large predefined kit (Milliplex canine cytokine panel, #CCYTMG-90K-PX13, Merck, Darmstadt, Germany) of 13 parameters: interleukin (IL)-2, IL-6, IL-7, IL-8, IL-10, IL-15, IL-18, tumour necrosis factor (TNF)-α, interferon (IFN)-γ, granulocyte-macrophage colony-stimulating factor (GM-CSF), IFN-γ–induced protein of 10 kd (IP-10), monocyte chemotactic protein-1 (MCP-1) and keratinocyte-derived chemokine (KC)-like. The method was performed according to the manufacturer’s instructions. All the samples were analysed in duplicate, and data were averaged.

### 2.8. Efficacy and Tolerance Assessment

The treatment success was evaluated by comparing the different scores at the beginning with the ones at the end of the study. The main parameter used for this was the cytological evaluation. Other parameters were a reduction in OTIS-3 and pruritus scores between D0 and D14. Immediate tolerance to the product was evaluated by the owner at each application and short- and medium-term tolerance by the evaluator through a general and dermatological examination. Signs of intolerance included immediate and prolonged discomfort of the animal, as well as signs of contact dermatitis (acute erythema and/or ulceration).

### 2.9. Statistical Analysis

All data were tested for normality distribution using the Shapiro-Wilk normality test. When data were normally distributed, homogeneity of variance was checked by Bartlett’s test and paired *t*-tests were performed. When data were not normally distributed at all-time points, nonparametric paired tests (Friedman or Mann–Whitney tests) were used after appropriate corrections if needed. *Ex æquo* values were corrected according to Hollander and Wolfe. Statistical analyses were performed using XLSTAT software (Microsoft^®^, version base 2020.4.1), and a two-sided *p*-value < 0.05 was considered statistically significant. 

## 3. Results

Twenty-one adult dogs were included in the study: twelve males and nine females. Breeds included Griffon (6), Basset (5), Bruno du Jura (4), Bleu de Gascogne (2), Border collie cross (1), Shar-peï (1), Beauceron (1) and shepherd cross (1). The median age of dogs was 4 years old [min 1–max 8]. Nineteen dogs presented with bilateral otitis externa, while the minority showed unilateral disease. The primary causes for most of the cases were not known, but six dogs suffered from atopic dermatitis. 

Three ears had an exudate score of 3, 20 a score of 2 and 17 showed a score of 1. According to the score, the ears were cleaned daily, every other day or biweekly, respectively. On average, 4.9 mL (standard deviation 2.1) of cleanser were used per cleaning.

### 3.1. Tolerance

All the animals completed the study. No adverse reaction was reported by the owners. No ototoxicity signs were observed. No abnormalities were detected during any of the clinical examinations at any time. 

### 3.2. Cytological Score

**Figure 1:**
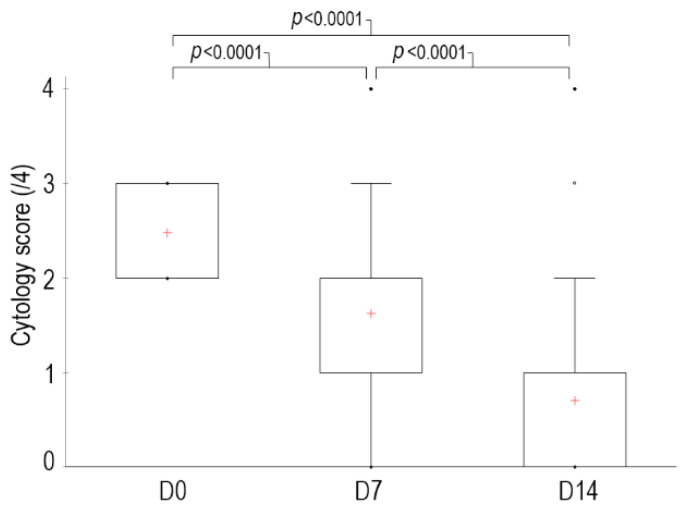
Kinetics of the global cytology score in dogs with erythemato-ceruminous otitis externa whose ears were cleaned with the tested ear cleanser at different time points during the study. Interpretation of the box and whiskers plots: the top of the reach box represents the third quartile, the red cross (+) in the middle is the mean, the bottom of the box is the first quartile and the upper and lower whiskers represent the minimum and maximum scores. Dots were considered as outliers.

The median global cytological score at D0 before the first cleaning was 2.5 out of 4 [2–3], with 21 ear canals with a score of 2 and 19 with a score of 3. At D7, the median score was reduced to 1.6 [0–4] (6 canals had a score of 0, 12 canals a score of 1, 14 a score of 2, 7 a score of 3 and 1 a score of 4) and at D14 it was reduced to 0.7 [0–4] (22 had a score of 0, 11 had a score of 1, 5 had a score of 2, 1 had a score of 3 and 1 a score of 4). The percentage of reduction in score was 34% at D7 and 73% at D14 (*p* < 0.0001) compared to D0 (Figure 1).

**Figure 2:**
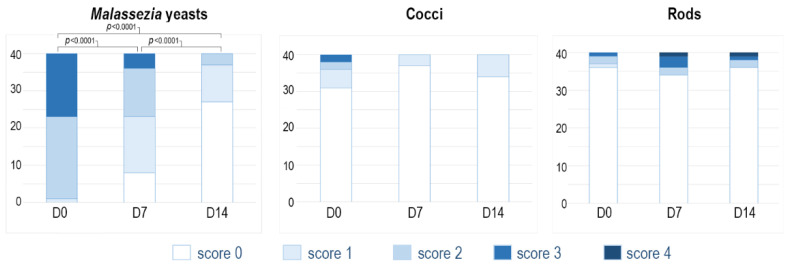
Stacked bar chart of the cytology score according to the type of microbe (yeasts, coccoid bacteria and rod-shaped bacteria) on Days 0, 7 and 14. The different intensities of colour in each bar show the number of ear canals with the different scores.

Concerning the different types of microbial agents (yeasts, coccoid bacteria and bacilli), the vast majority of samples initially corresponded to a proliferation of yeasts (Figure 2), with scores of 2 (22 samples) or 3 (17 samples). 

The median score at D0 was 2 [1–3], which was significantly reduced to 1 on D7 (*p* < 0.0001) and subsequently to 0 on D14 (*p* < 0.0001). Samples with coccid or rod-shaped bacteria visible on cytology were too few to enable interpretation of the data.

### 3.3. OTIS-3 Score

**Figure 3:**
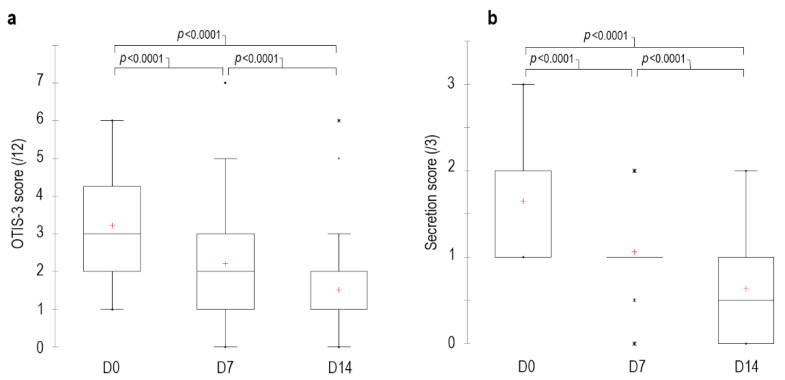
Kinetics of the OTIS-3 (**a**) and secretion (**b**) scores in dogs with erythemato-ceruminous otitis externa cleaned with the tested ear cleanser at different time points during the study. Explanation for the box and whisker plots: the top of each box represents the third quartile, the red cross (+) in the middle is the mean, the horizontal bar in the box is the median, the bottom of the box is the first quartile and the upper and lower whiskers represent minimum and maximum scores. Dots were considered as outliers.

The median OTIS-3 score on D0 before the first clean was 3 [min 1–max 6], 2 [0–7] on D7 and 1 [0–6] on D14. The percentage of reduction in score was 31% at D7 and 53% at D14 (*p* < 0.0001) compared to D0 (Figure 3). The OTIS-3 score of 33 canals decreased between D0 and D14, the score of 5 canals remained stable and the score of 2 canals increased (3 out of 12) on D14 compared to D0 (2 out of 12). On D14, 36 ears had a secretion score ≤1, and the remaining 4 ears had a score of 2 out of 3. Finally, a 48% reduction in erythema was observed on D14 compared to D0 (*p* < 0.0001).

### 3.4. Pruritus Score

On D0, the median pruritus score before the first application was 0.8 [0.2–8.6], 0.1 [0–9.8] on D7 and 0.1 [0–7.2] on D14. The percentage reduction in the score was 40% at D7 and 60% at D14 (*p* < 0.001) compared to at D0.

### 3.5. Cytokines and Lipids

**Figure 4:**
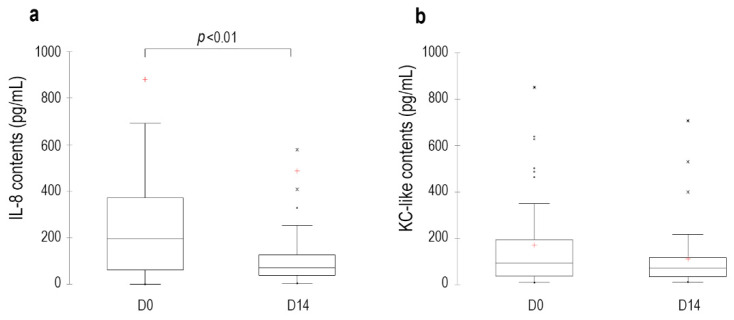
Kinetics of the IL-8 (**a**) and KC-like (**b**) contents in dogs with erythemato-ceruminous otitis externa cleaned with the tested ear cleanser at different time points during the study. Explanation for the box and whisker plots: the top of the box represents the third quartile, the red cross (+) is the mean, the horizontal bar in the box is the median, the bottom of the box is the first quartile and the upper and lower whiskers represent minimum and maximum scores. Dots were considered as outliers.

On D0, among the 13 markers evaluated, only IL-8 and KC-like were detectable. On D14, their content was reduced compared to baseline (IL-8: −45%, *p* < 0.01; −36%, KC-like: *p* = 0.3, respectively) (Figure 4). Other cytokines did not significantly change compared to baseline or too many values were below the detection threshold.

**Figure 5:**
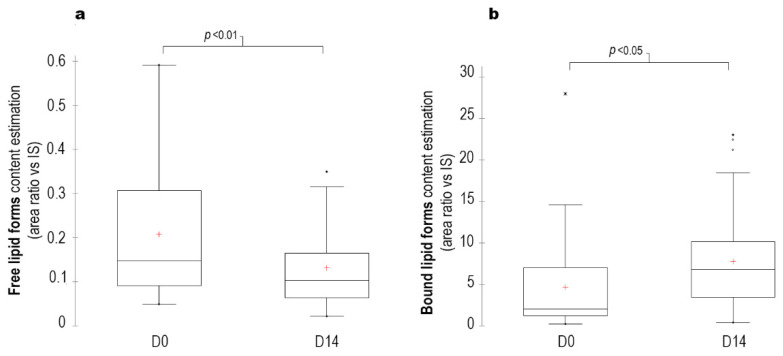
Kinetics of free lipid forms (**a**) and of bound lipid forms (**b**) concentrations in dogs with erythemato-ceruminous otitis externa that received the tested ear cleanser at different time points during the study (results are expressed as area ratio vs internal standard (IS)). Explanation for box and whisker plots: the top of the box represents the third quartile, the red cross (+) is the mean, the horizontal bar in the box is the median, the bottom of the box is the first quartile and the upper and lower whiskers represent minimum and maximum scores. Dots were considered as outliers.

The lipid profile also changed, free lipids (fatty aldehydes, fatty acids and fatty alcohols) decreased by 40% (*p* < 0.01), whereas bound lipids (waxes, triglycerides, short- and long-chain cholesterol esters) increased by 65% (*p* < 0.05) at D14 (Figure 5).

### 3.6. Bacterial and Fungal Culture Results

#### 3.6.1. Bacterial Cultures

Samples were collected from 40 ears for bacterial culture at D0, but only 39 could be used due to a technical problem with one sample. Positive bacterial cultures (19/39) mainly yielded *Clostridium perfringens* (7/39), *Staphylococcus pseudintermedius* (6/39) and *Pseudomonas aeruginosa* (4/39). 

Samples were collected from 40 ear canals at D14. Positive bacterial cultures (15/40) yielded a scanty polymicrobial culture (2/40), *S. pseudintermedius* (6/40), *Enterobacter gergoviae* (2/40), *P. aeruginosa* (4/40), *Burkholderia multivorans* (1/40), *Enterobacter* spp. (1/40) and *C. perfringens* (2/40). Two ear canals had multibacterial flora: one yielded *P. aeruginosa* with *S. pseudintermedius* and *B. multivorans* and the second one *P. aeruginosa* and *Enterobacter* spp. Three cultures that were negative at D0 were positive at D14 with rare colonies of *S. pseudintermedius*.

#### 3.6.2. Fungal Cultures

For fungal cultures, due to unforeseen laboratory reasons, samples were only cultured from 20 ears at D0. Positive fungal cultures (8/20) yielded *M. pachydermatis* (5/20) and *Candida* spp. (3/20).

At D14, 32 ears were sampled. Positive fungal cultures (5/32) generated *C. albicans* (1/32) and *M. pachydermatis* (4/32).

#### 3.6.3. Comparison between Cytology and Culture Results

A total of 79 bacterial cultures and 52 fungal cultures were done during the study. The results of the cytology examination and the bacterial culture were in agreement in 59 out of 79 samples (75%) for cocci, 60 out of 79 samples (76%) for rods and 21 out of 52 samples (40%) for yeasts (Table 1).

Cytology examination underestimated the presence of cocci in 9 out of 79 (11%) samples, of rods in 18 out of 79 (23%) samples and of yeast in 11 out of 52 (21%) samples (Table 1).

## 4. Discussion

The significant decrease in the secretion score observed in this study is in agreement with the results of previous studies describing the efficacy of this ear cleaner in reducing the amount of cerumen in the ear canal both in vivo and in vitro [25,26]. In addition to its antiseptic action, the ceruminolytic effect helps to remove bacterial toxins, cell debris and free fatty acids that could serve as stimuli for further inflammation [8,27]. The OTIS-3 score decreased significantly by 53%, with a 48% reduction in erythema at D14. These data illustrate the role of inflammation as a primary, often self-perpetuating, factor in otitis externa [5]. These figures are supported by the significant decrease in the amount of IL-8 detected at the surface of the auditory canals. IL-8 is a potent chemoattractant for neutrophils and a proinflammatory mediator. It has been shown to be significantly elevated in some atopic dogs [1], including ear canals [28] and conjunctivae [29], but also in ear canals infested with *O. cynotis* [28]. Its 45% reduction compared to baseline is likely linked to the decrease in aural inflammation. KC-like is a chemoattractant for neutrophils [30] that has been shown to be present in increased quantities in dogs affected by babesiosis, sepsis, urinary tract infection or trauma [31,32,33]. Its decrease at D14, similar to that of IL-8, is likely linked to the reduction in ear canal inflammation. In this study, the pruritus score was low at inclusion (0.8 out of 10). Although the reduction was significant after the ear cleaner application, no clinical conclusion can be drawn.

In the present study, the use of the ear cleaner was able to significantly and effectively reduce fungal content over a 14-day period. The bacterial content was also reduced, but the overall bacterial load was initially low, which makes it difficult to draw firm conclusions.

The cytological score decreased by 72% (*p* < 0.0001) between D0 and D14. This confirms the results of the bacterial and fungal cultures. The three samples that initially tested negative and subsequently tested positive for *S. pseudintermedius* at D14 could be part of the normal flora as a positive culture does not necessarily imply clinical disease [34]. However, in the other four ears that had *P. aeruginosa* cultured at D0 and rods observed on the cytology, the population was identical at D14. *Pseudomonas* are ubiquitous organisms that tend to prefer a wet environment. They are generally opportunistic pathogens commonly involved in chronic otitis, which could have been the case in these dogs [1]. 100% bactericidal activity against all strains of *P. aeruginosa* and *S. pseudintermedius* was previously shown for the tested ear cleaner in vitro [26]. The apparent lack of effect in these dogs could be due to the absence of a topical or systemic corticosteroid, which is of value when atopic dermatitis is the underlying cause, or to the absence of a topical antibiotic. Lastly, the antiseptic effect could be impaired by the presence of cerumen or antiadhesive or other nonkilling mechanisms [25].

The significant decrease in *M. pachydermatis* may be attributed to the antiseptic effect of the ear cleaner, which has been shown to be 100% effective in vitro, or to its ceruminolytic effect or a combination thereof [26]. Large quantities of fatty acids are often found in the ears of dogs with otitis externa and are used by *M. pachydermatis* as their preferred medium for growth [35], although most strains are traditionally described as nonlipid dependent [36]. The decrease in the amount of free fatty acids at D14 could be due to the antiseptic effect of the product, as bacterial degradation of lipids generally results in more free fatty acids [37] or simply results from the ceruminolytic activity and mechanical removal of the product.

Two ear cleaners containing 2.5% lactic acid and 0.1% salicylic acid have shown good activity against *S. intermedius*, *P. aeruginosa*, *Proteus* spp. and *M. pachydermatis* in vitro and in vivo [9,10,12]. An in vitro study comparing the antimicrobial efficacy of a 2.5% lactic acid and 0.1% salicylic acid ear cleaner and the product, which was evaluated in our study, found that both ear cleaners were 100% bactericidal against *S. pseudintermedius* and *P. aeruginosa* but that only the tested ear cleaner was effective against *M. pachydermatis* [26]. It also achieved a knockout effect at one minute for all three pathogens tested, while the 2.5% lactic acid and 0.1% salicylic acid ear cleaner achieved a knockout effect at eight minutes for *S. pseudintermedius* and at four minutes for *P. aeruginosa* [26]. The cleaning performance of the tested ear cleaner was also better than the one containing 2.5% lactic acid and 0.1% salicylic acid [25].

The antibacterial activity of several other ear cleaners has also been demonstrated in vitro [10,15,20,38]. However, it is difficult to compare the various products for their antimicrobial efficacy due to their different ingredients and component concentrations [20]. Cleaners containing alcohol may be more effective than cleaners with no alcohol, as alcohols rapidly kill most organisms. The drawbacks of cleaners containing alcohol include possible pain and irritation, especially in inflamed ears [8]. The isopropyl alcohol contained in the tested product is an astringent which has a potential antimicrobial effect thanks to its drying effect on the ear canal [8].

The tested ear cleaner also contains lipacids, which have been shown to have a similar bacteriostatic activity in vitro to that of benzoyl peroxide on germs involved in human acne such as *S. aureus*, *S. epidermidis* and *Propionibacterium acnes* [39]. Traditionally, it has been considered that an acid pH is associated with antibacterial activity, but more recent studies suggest the antimicrobial action of ear cleaners is not pH dependent [20,38]. In the present study, the neutral pH of the ear cleaner did not seem to have a detrimental effect on its antimicrobial performance.

The design of the study (open, uncontrolled) can be considered a limitation. If the use of a placebo product is not ethically suitable, a ceruminolytic product as a positive control could be considered. Nevertheless, the objective measurement of inflammatory markers, as well as cytological examinations and microbial cultures, performed by persons not involved in the clinical evaluation, may partly compensate for the lack of clinical blinding. The cohort recruited did not enable a robust evaluation of the effectiveness of the cleanser in bacterial proliferation. Further studies, targeting otitis externa with a substantial bacterial component, are desirable. Tympanic membrane integrity was assessed during video-otoscopy, whenever possible, i.e., when the amount of secretion was low enough to allow a correct visualisation. No ototoxicity signs were observed. Nevertheless, in the absence of specific studies, the use of this ear cleaner is not recommended in case of a ruptured eardrum.

The methods used for microbiological diagnosis in the present study were conventional (cytology, swab and culture). The low number of positive fungal cultures compared to positive cytological examination (20/52, 38.4%) is surprising and unusual [40]. We have no explanation for this low detection by culture. Recent data on ear microbiota reveal the wide range of germs present in otitis and in apparently healthy canals [41,42,43,44]. The techniques used in the present study are those used by veterinarians in their daily practice. Veterinarians will be able to appropriate the results more easily than results obtained by 16s rRNA, although from a fundamental knowledge point of view, it would have been interesting to also have conducted an investigation using 16s RNA.

Finally, the follow-up was carried out for only two weeks. Long-term use, particularly in the case of recurrent atopic otitis, should be assessed in order to monitor the product’s benefit in preventing or reducing the frequency of relapses.

## 5. Conclusions

The tested ear cleaner, used at a frequency based on the amount of ear canal secretions, significantly reduced the infectious and inflammatory components of ECOE and can thus be used as an additional treatment option.

## Figures and Tables

**Table 1:** Contingency table showing the results of cytology and bacterial and fungal cultures performed on dogs with erythemato-ceruminous otitis externa cleaned with the tested ear cleanser. Results obtained on D0 and D14 are pooled.

	Coccoid Bacteria		Rod-Shaped Bacteria		Yeasts	
	Positive Culture	Negative Culture	Positive Culture	Negative Culture	Positive Culture	Negative Culture
Positive cytology	4/79	11/79	7/79	1/79	2/52	20/52
Negative cytology	9/79	55/79	18/79	53/79	11/52	19/52

## Data Availability

The data presented in this study are available from the corresponding author upon request. The data are not publicly available due to the need to maintain patient confidentiality.
